# The regulation of combined treatment-induced cell death with recombinant TRAIL and bortezomib through TRAIL signaling in TRAIL-resistant cells

**DOI:** 10.1186/s12885-018-4352-3

**Published:** 2018-04-16

**Authors:** Sunhyo Ryu, Yun Jeong Ahn, Chakeong Yoon, Jeong Hwan Chang, Yoonkyung Park, Tae-Hyoung Kim, Amanda R. Howland, Cheryl A. Armstrong, Peter I. Song, Ae Ran Moon

**Affiliations:** 1 Department of Dermatology, University of Colorado Denver School of Medicine, Aurora, CO 80045 USA; 2 Department of Biomedical Science and Research Center for Proteinaceous Materials, Chosun University School of Medicine, 309 Pilmoon-Daero, Gwangju, 61452 Republic of Korea; 3 Department of Biomedical Science and Research Center for Proteinaceous Materials, Chosun University, Gwangju, South Korea; 4 Department of Surgery, Chosun University School of Medicine, Gwangju, South Korea; 5 Department of Biomedical Science and Research Center for Proteinaceous Materials, Chosun University, Gwangju, South Korea; 6 Department of Biochemistry, Chosun University School of Medicine, Gwangju, South Korea; 7 Present Address: Cheomdan Medical Center, 170 Cheomdanjungang-ro, Gwangsan-gu, Gwangju, 62276 Republic of Korea; 8 Department of Dermatology, University of Colorado Denver Anschutz Medical Campus, 12801 E. 17th Avenue, Aurora, CO 80045 USA

**Keywords:** Recombinant TRAIL, Bortezomib, Combination treatment, Caspase-dependent, Apoptosis

## Abstract

**Background:**

Multiple trials have attempted to demonstrate the effective induction of cell death in TRAIL-resistant cancer cells, including using a combined treatment of recombinant TRAIL and various proteasome inhibitors. These studies have yielded limited success, as the mechanism of cell death is currently unidentified. Understanding this mechanism’s driving forces may facilitate the induction of cell death in TRAIL-resistant cancer cells.

**Methods:**

Three kinds of recombinant soluble TRAIL proteins were treated into TRAIL-resistant cells and TRAIL-susceptible cells, with or without bortezomib, to compare their respective abilities to induce cell death. Recombinant TRAIL was treated with bortezomib to investigate whether this combination treatment could induce tumor regression in a mouse syngeneic tumor model. To understand the mechanism of combined treatment-induced cell death, cells were analyzed by flow cytometry and the effects of various cell death inhibitors on cell death rates were examined.

**Results:**

ILz:rhTRAIL, a recombinant human TRAIL containing isoleucine zipper hexamerization domain, showed the highest cell death inducing ability both in single treatment and in combination treatment with bortezomib. In both TRAIL-resistant and TRAIL-susceptible cells treated with the combination treatment, an increase in cell death rates was dependent upon both the dose of TRAIL and its intrinsic properties. When a syngeneic mouse tumor model was treated with the combination of ILz:rhTRAIL and bortezomib, significant tumor regression was seen as a result of the effective induction of cancer cell death. The combination treatment-induced cell death was both inhibited by TRAIL blocking antibody and caspase-dependent. However, it was not inhibited by various ER stress inhibitors and autophagy inhibitors.

**Conclusions:**

The combination treatment with ILz:rhTRAIL and bortezomib was able to induce cell death in both TRAIL-susceptible and TRAIL-resistant cancer cells through the intracellular TRAIL signaling pathway. The efficiency of cell death was dependent on the properties of TRAIL under the environment provided by bortezomib. The combination treatment-induced cell death was not regulated by bortezomib-induced ER stress response or by autophagy.

**Electronic supplementary material:**

The online version of this article (10.1186/s12885-018-4352-3) contains supplementary material, which is available to authorized users.

## Background

Tumor necrosis factor-related apoptosis-inducing ligand (TRAIL) was first identified in 1996 [1]. TRAIL induces caspase-dependent apoptosis in cancer cells after binding to its corresponding receptors: DR4 or DR5 in humans and DR5 in rodents. Due to its cancer cell specific death-inducing activity, TRAIL has been regarded as a potential protector or inhibitor of tumor generation and progression [2, 3]; however, lots of tumor cell lines and primary cancer cells are resistant to TRAIL-induced apoptosis [4–6]. Additionally, TRAIL activates non-apoptotic signals, including NF-kB, mitogen activated kinase (MAPK), JNK, p38, Akt, and ERK [7–13]. TRAIL actually induces the proliferation of vascular endothelial cells and specific cancer cells, rather than inhibiting them [14–16]. These potential adverse effects accelerate research interest regarding the resistance mechanism of TRAIL-induced apoptosis and in the potential to combine TRAIL with an additional agent to induce apoptosis [17–22].

Research to induce cell death in TRAIL resistant cells has demonstrated some success. TRAIL induced apoptosis has been achieved through combined treatment with certain chemical agents, as well as through the genetic modification and subsequent expression of certain apoptosis related proteins [4, 7, 17, 18, 23–33]. Though regulating specific apoptosis-related proteins can induce cell death, this process is not applicable to all TRAIL-resistant cells because the resistance mechanisms are heterogeneous. According to published reports, several additional agents can be employed to induce apoptosis via TRAIL. Some agents regulate the balance between pro-apoptotic and anti-apoptotic proteins, while others modify membrane lipid composition to provide more opportunities for DR4 or DR5 to localize to membrane lipid rafts in specific cells. [26, 34, 35]. Many researchers have found that apoptosis-related proteins involved in the TRAIL signaling pathway do not carry any mutations. Both the expression and membrane localizations of TRAIL receptors are well regulated in almost all TRAIL resistant cells.

In this study, we hypothesized that specific intracellular conditions would be required to induce cell death in TRAIL resistant cancer cells and that these conditions could be provided by an additional agent, such as bortezomib. In addition, the effective cell death inducing ability of recombinant TRAIL would be essential. We tested various recombinant TRAIL proteins to compare cell death inducing ability; either single treatment in TRAIL-susceptible cells, or combined treatment with bortezomib in TRAIL-resistant cells. Tumor progression was inhibited with the combined treatment of bortezomib and ILz:rhTRAIL; the latter being a recombinant human TRAIL protein containing isoleucine zipper hexamerization motif for efficient multimerization. The cell death induced by the combined treatment was found to be influenced by TRAIL blocking antibody and regulated by caspases. It was not affected by ER stress or autophagy.

## Methods

### Cell culture

CT26 (80009), B16F10 (80008), MDA-MB-231 (30026), and HEK 293 (21573) cells were purchased from the Korean Cell Line Bank (KCLB, Seoul, Korea). HeLa (HC18802) and Jurkat (HC18111) cells were purchased from the Korean Collection for Type Cultures (KCTC, Daejeon, Korea). 4 T1 (CRL-2539) cells were purchased from the American Type Culture Collection (ATCC). BMK (baby mouse kidney) cells were kindly provided by Dr. J. Hiscott. The cells were cultured, according to the recommendations of KCTC, in Gibco™ Dulbecco’s Modified Eagle Medium (DMEM) or RPMI-1640 (Thermo Fisher Scientific, Pittsburg, PA, USA) supplemented with 5% fetal bovine serum (Capricorn Scientific, Ebsdorfergrund, Germany) and 1% Gibco™ penicillin-streptomycin at 37 °C in a humidified atmosphere containing 5% CO_2_.

### XTT assay

Cell viability was measured by XTT assay using Cell Titer 96® Aqueous one solution reagent (Promega, Madison, WI, USA), according to the manufacture’s protocol. Cells were seeded into a 96-well plate (1 × 10^4^ cells/well) a day before treatment with recombinant TRAIL and/or bortezomib, which was purchased by Selleck Chemicals (Houston, TX, USA). The Medium was changed to phenol-red free DMEM or RPMI with 10% of FBS and then treated with recombinant TRAIL and/or bortezomib. After an appropriate time period, substrate solution in the kit was added into the medium, and optical density was measured at 490 nm using a microplate reader (Infinite M200™; Tecan, Männedorf, Switzerland). Data are presented as the relative percentage of viable cells using the average optical density (O.D.) of the untreated control as a reference (100%) in each experiment.

### Preparation of cell lysates

Cell lysates were prepared for immunoblotting analysis. Cells were harvested with ice-cold RIPA buffer (50 mM Tris-HCl pH 7.4, 1% NP-40, 0.5% Na-deoxycholate, 0.1% SDS, 150 mM NaCl, 2 mM EDTA) and supplemented with phosphatase inhibitor cocktails-I, -II (Merck Millipore, Darmstadt, Germany). Proteins were quantified using Pierce™ BCA (bicinchoninic acid) protein assay kit (Thermo Fisher Scientific). Protein samples were loaded onto SDS-PAGE gel and transferred to Polyvinylidene difluoride (PVDF) membrane (Amersham, Buckinghamshire, England).

### Immunoblotting analysis and antibodies

To examine expression levels of specific proteins, immunoblotting analysis was performed using appropriate antibodies. For immunoblotting, the membranes were immersed for 1 h into Tris-buffered saline (TBS), containing 5% skim milk (Thermo Scientific), then incubated with appropriate primary antibodies. After 3 h of incubation at room temperature or overnight at 4 °C, the membrane was washed with 0.05% TBST solution (TBS with 0.05% Tween-20) three times for 10 min each and incubated for 1 h with horseradish peroxidase-conjugated secondary antibody (Jackson ImmunoResearch, West Grove, PA, USA). After extensive washing, immunoreactive proteins were detected using PowerOpti-ECL™ solution (Animal Genetics, Suweon, Korea). Intensities of reactive bands were measured and compared using Image J software (www.ImageJ.net).

### Antibodies

Antibodies were used to neutralize TRAIL signaling and for immunoblotting analysis. Anti-TRAIL antibody (B-T24) for neutralization was purchased from Diaclone (Besançon cedex, France). For immunoblotting analysis, antibodies against caspase 3 (9662), caspase 9 (9508), Bcl-2 (2876), Bcl-XL (2764), and Mcl-1 (5453) were purchased from Cell Signaling Technology (Danvers, MA, USA). Anti-caspase-8 (ALX-804-477) antibody was purchased from Enzo Life Sciences (Farmingdale, NY, USA) and anti-β-actin antibody (MAB1501) was purchased from Millipore (Thermo Fisher Scientific). Anti-XIAP antibody (sc-55,551) was purchased from Santa Cruz Biotechnology Inc. (Santa Cruz, CA, USA).

### Recombinant TRAIL proteins

Recombinant human and mouse TRAIL proteins (rhTRAIL and rmTRAIL) were purchased from PeproTech (PeproTech Korea, Seoul, South Korea): rhTRAIL (310–04); rmTRAIL (315–19). ILz:rhTRAIL was constructed and purified by Dr. Kim, as described in a previous report [36]. Briefly, hexamerization isoleucine zipper is fused to the N-terminal of recombinant human TRAIL (amino acid 114–281) resulting in ILz:rhTRAIL. The recombinant ILz:rhTRAIL protein was expressed in *E. coli* BL21 (DE3) and purified using a Ni-NTA His affinity column (Novagen, Carlsbad, CA, USA) according to the manufacturer’s instructions.

### Flow cytometry

Cells were treated with ILz:rhTRAIL and bortezomib, with or without pre-treatment with z-VAD-fmk for 1 h. After 24 h of treatment with ILz:rhTRAIL and bortezomib, cells were harvested by trypsin treatment and stained with propidium iodide (PI) and Annexin V using the FITC Annexin V Apoptosis Detection Kit (BD Biosciences, Thermo Fisher Scientific) for 5 min. Stained cells were analyzed by BD FACS Calibur™ analyzer and the BD CellQuest™ program (BD Biosciences, Thermo Fisher Scientific).

### Animal experiment

A syngeneic tumor model was generated to analyze whether the combined treatment of TRAIL with bortezomib could have an effect on tumor regression. Cultured CT26 cells were harvested and re-suspended with phosphate-buffered saline (PBS) following trypsin treatment. Sixty Balb/c mice, provided by Orient Bio (Sungnam, Korea), were divided into 4 groups: ‘sham’, ‘bortezomib’, ‘TRAIL’, and ‘TRAIL and bortezomib’. Cells were subcutaneously injected into the backs of all seven-week old Balb/c mice (2 × 10^5^ cells/mouse), except for those mice in the ‘sham’ group.The size of each tumor was measured using a caliper (CD-15CPX, Mitutoyo, Japan) and tumor volume was calculated according to the equation: tumor volume = (L × W × W)/2, L = length, W = width. Ten days following the subcutaneous injection of cells, when average tumor volumes around 40 mm^3^ were reached, ILz:rhTRAIL (10 μg/kg) and/or bortezomib (3.8 μg/kg) were intravenously injected via tail vein. This consisted of a series of 5 injections, with a two-day interval between each injection. After these 5 injections were completed, tumor tissues were harvested, fixed with 10% formalin solution, and embedded with paraffin. Animal experiments were performed at Chosun University in accordance with the guidance of Chosun University Institutional Animal Care and Use Committee (acceptance number: CIACUC2016-A0023).

### Statistically analysis

Statistical significance was determined by Student’s *t*-test or ANOVA single test. A two-tailed *p* < 0.05 was considered statistically significant.

## Results

### ILz:rhTRAIL’s superiority in inducing cell death in TRAIL-susceptible cells

**Fig. 1:**
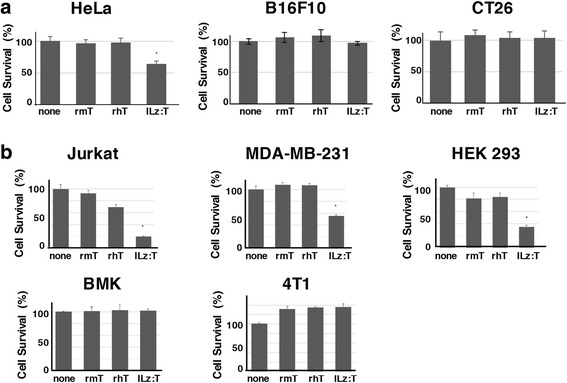
ILz:rhTRAIL showed the highest cell death inducing ability in TRAIL susceptible cells. Indicated cells were cultured onto a 96-well plate and treated with varying recombinant TRAIL proteins: rmT, recombinant mouse TRAIL from Peprotech (rmTRAIL); rhT, recombinant human TRAIL from Peprotech (rhTRAIL); ILz:T, isoleucine zipper hexamerization motif containing recombinant human TRAIL. (**a**) HeLa, CT26, and B16F10 cells were treated with varying recombinant TRAIL proteins (100 ng/ml). After 16 h, cell survival was examined by XTT assay. (**b**) Three human cell lines (Jurkat, MDA-MB-231, and HEK 293) and two murine cell lines (BMK and 4 T1) were treated with varying recombinant TRAIL proteins (100 ng/ml). To investigate TRAIL-susceptibility, cell death was analyzed by XTT assay 24 h after treatment. The relative values of the XTT assay were examined after comparing the results to untreated controls. *p < 0.05, compared with untreated controls by Student’s *t*-test

To identify the effect of TRAIL on cell death inducing ability, we examined the cell death rates induced by three soluble recombinant TRAIL proteins in various mouse or human cells: recombinant mouse TRAIL (rmTRAIL), recombinant human TRAIL (rhTRAIL), and isoleucine zipper hexamerization motif containing recombinant human TRAIL (ILz:rhTRAIL). The rmTRAIL and rhTRAIL used in this study either contain an extracellular domain of mouse or human TRAIL, respectively. The ILz:rhTRAIL, which contains an extracellular domain of human TRAIL and an isoleucine zipper hexamerization motif, demonstrated greater formation of multimers which in turn enhances its cell death inducing ability [36]. According to the previous report, ILz:rhTRAIL is a pre-multimerized recombinant TRAIL and facilitates caspase activation, tBid generation, and chromatin condensation [36]. The researchers speculated that ILz:rhTRAIL could stimulate multimerization of DR5 on the cell membrane to induce high levels of cytotoxicity. Treatment with 100 ng/ml ILz:rhTRAIL for 16 h induced cell death in 40% of HeLa cells; in contrast, treatment with either rmTRAIL or rhTRAIL failed to demonstrate any significant cell death in HeLa cells (Fig. 1a), despite their known susceptibility to TRAIL-induced apoptosis [37]. The cell death of CT26 colon cancer cells and B16F10 melanoma cells was not induced by the three recombinant TRAILs used in this study (Fig. 1a).

To investigate whether the cells were susceptible or resistant to TRAIL-induced cell death, three human cells (Jurkat, MDA-MB 231 and HEK 293) and two mouse cells (BMK and 4 T1) were treated with three recombinant TRAIL proteins (Fig. 1b). Human leukemia cells (Jurkat cells) were susceptible to cell death in a dose-dependent manner when treated with rhTRAIL and ILz:rhTRAIL (Additional file 1: Figure S1). ILz:rhTRAIL induced cell death in human breast cancer MDA-MB-231 cells. The cell death of transformed human embryonic kidney cells (HEK 293) with sheared human adenovirus 5 DNA was induced by ILz:rhTRAIL treatment, while in contrast, cell death was only minimally seen in treatment with rhTRAIL. Furthermore, all three recombinant TRAIL proteins failed to cause any significant cell death in transformed baby mouse kidney BMK cells and mouse mammary tumor 4 T1 cells. Taken together, these results demonstrate ILz:rhTRAIL’s superiority in inducing cell death in TRAIL susceptible cells: HeLa, Jurkat, MDA-MB-231, and HEK 293.

### ILz:rhTRAIL’s superiority in the combined treatment-induced cell death with bortezomib in TRAIL resistant cells

Since only four out of eight cell lines were killed by recombinant TRAIL in Fig. 1, TRAIL was combined with an additional agent to induce cell death in TRAIL-resistant cells. Proteasome inhibitors are frequently used for combined treatment with TRAIL, therefore, we used several proteasome inhibitors as adjuvants with TRAIL in CT26 and B16F10 cells [20, 38]. Bortezomib was the best additional agent to combine with TRAIL to induce cell death (data not shown). Although bortezomib induced cell death in four TRAIL resistant cell lines, cell death rates were less than 40% after 24 h, even with 100 nM of bortezomib (Additional file 2: Figure S2). Considering that the cell death rate is over 50% in bortezomib-susceptible cells treated with 10 to 50 nM of bortezomib for 24 h, these four cell lines seemed to be either completely resistant or moderately resistant to bortezomib-induced cell death [39].

**Fig. 2:**
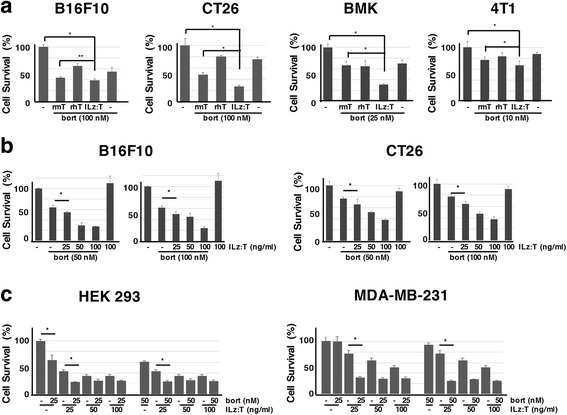
ILz:rhTRAIL showed the highest cell death inducing ability in the combination treatment of recombinant TRAIL and bortezomib. Cells were cultured onto 96-well plates with 80% to 90% of confluency. Cell death rates were analyzed by XTT assay 24 h after the indicated treatment. The relative values of the XTT assay were examined following comparison to untreated controls. (**a**) Each cell was treated with 100 ng/ml of recombinant TRAIL protein and a distinctive amount of bortezomib as indicated: rmT, recombinant mouse TRAIL from Peprotech (rmTRAIL); rhT, recombinant human TRAIL from Peprotech (rhTRAIL); ILz:T, isoleucine zipper hexamerization motif containing recombinant human TRAIL (ILz:rhTRAIL). (**b**) Two TRAIL-resistant cell lines (B16F10 and CT26) were treated with fixed amounts of bortezomib (50 and 100 nM) and serially increasing amounts of ILz:rhTRAIL. (**c**) Two TRAIL-sensitive cell lines (HEK 293 and MDA-MB-231) were treated with fixed amounts of bortezomib (25 and 50 nM) and serially increasing amounts of ILz:rhTRAIL: bort, bortezomib. * and **p < 0.05 and *p* < 0.1 by Student’s *t*-test, respectively

Since the three recombinant TRAIL proteins demonstrated different cell death inducing abilities in TRAIL-susceptible cells, we decided to examine the cell death inducing ability of the combined treatment. The three different recombinant TRAIL proteins were all separately combined with bortezomib, in TRAIL resistant cells. (Fig. 2a). Individual cells were then treated with a consistent amount of recombinant TRAIL protein (100 ng/ml) and a minimal amount of bortezomib. With this approach, cell death rates were no longer dose-dependently increased as illustrated in Additional file 2: Figure S2. XTT assay demonstrated that after 24 h of treatment, over 50% of cells were killed with the combined treatment of ILz:rhTRAIL and bortezomib in three TRAIL-resistant cell types (B16F10, CT26, and BMK) (Fig. 2a). Though only about 30% of 4 T1 cells were killed with the combined treatment of ILz:rhTRAIL and bortezomib, this could still be significant considering that 4 T1 cells tended to proliferate with a single treatment of TRAIL, as seen in Fig. 1 and Additional file 3: Figure S3. Because ILz:rhTRAIL demonstrated the highest cell death inducing ability in both TRAIL-resistant cells treated with the combination therapy, and TRAIL-sensitive cells treated with ILz:rhTRAIL alone, we used ILz:rhTRAIL in further examinations of this report.

To identify the major determinant of cell death, death rates were examined following treatment with varying amounts of ILz:rhTRAIL and bortezomib (Fig. 2b, c). In TRAIL resistant B16F10 and CT26 cells, cell death rates rose dose-dependently following treatment with a fixed dose of bortezomib and increasing amounts of ILz:rhTRAIL (Fig. 2b). Cell death rates did not significantly increase when the dose of bortezomib was doubled, but the amount of ILz:rhTRAIL remained constant. These results were the same in TRAIL susceptible HEK 293 and MDA-MB 231 cells (Fig. 2c). The cell death rates observed with the combined treatment were both dependent on the dose of recombinant TRAIL protein, and regulated by its intrinsic properties.

### Tumor regression induced by the combined treatment of ILz:rhTRAIL with bortezomib in a syngeneic mouse tumor model

**Fig. 3:**
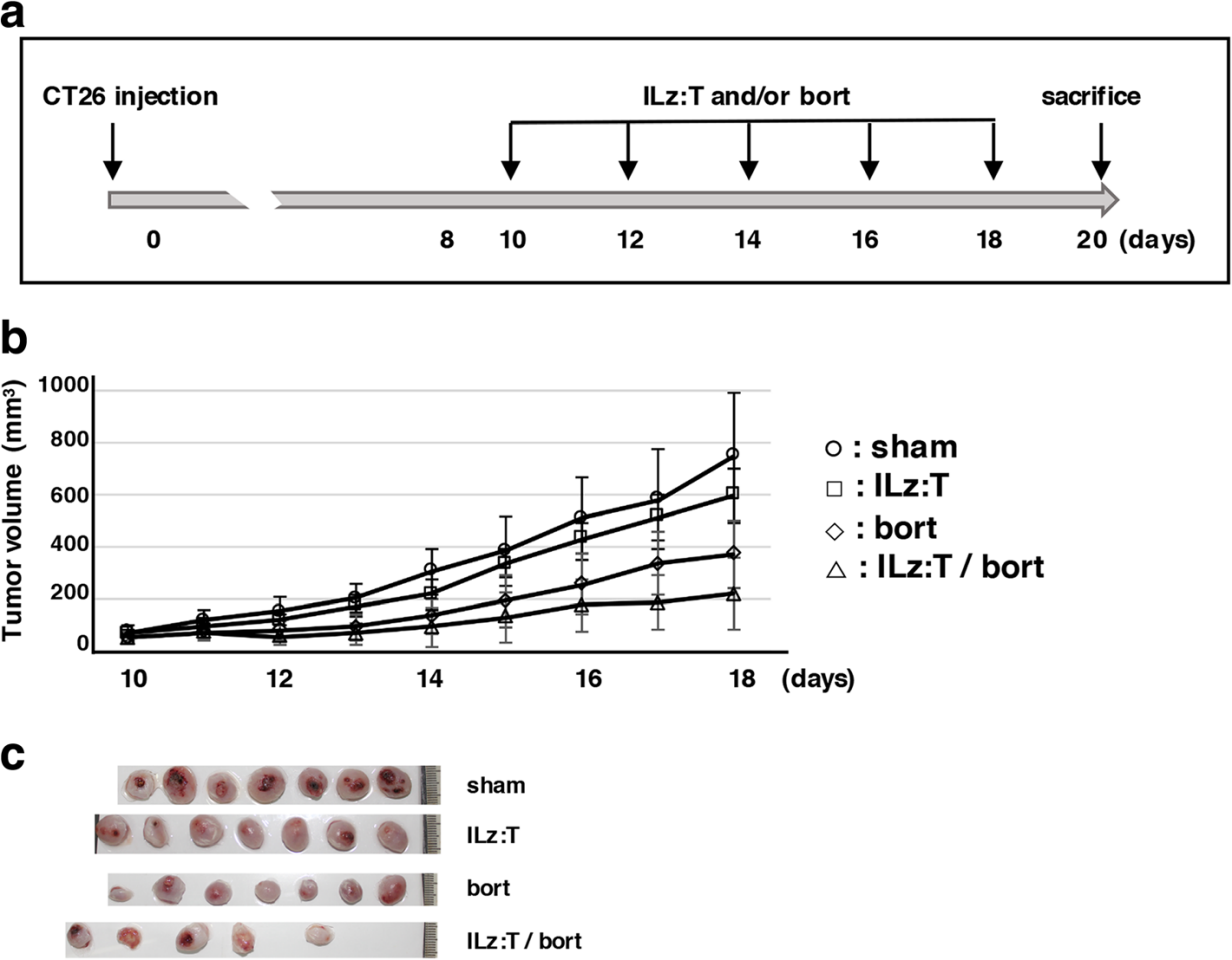
Tumors regressed with the combination treatment in a syngeneic mouse tumor model. CT26 cells (2 × 10^5^ / mice) were subcutaneously injected into the backs of seven-week old Balb/c mice. A total of 60 mice were used in this experiment: 15 mice per group. ILz:rhTRAIL and/or bortezomib were injected via the tail vein every 2 days from 10 days after the injection of CT26 cells, when average tumor volume reached 40 mm^3^. The ‘sham control’ mice were injected with phosphate-buffered saline, ‘ILz:T’ mice were injected with ILz:rhTRAIL (10 μg/ kg), ‘ILz:T/bort’ mice were injected with ILz:rhTRAIL (10 μg/ kg) and bortezomib (3.8 μg/ kg), and ‘bort’ mice were injected with bortezomib (3.8 μg/ kg). Tumor volumes were measured from the day that CT26 cells were injected until mice were sacrificed. Mice were sacrificed 20 days after being injected with CT26 cells. The experimental time table is depicted in (**a**). (**b**) Average tumor volumes in each group prior to sacrifice are represented, which were recorded from day 10 to day 18. Statistical significance for the four groups was identified by ANOVA single test (*p* < 0.05). (**c**) Tumors were isolated and pictures taken before formalin fixation. Although seven mice were represented in each group, only five tumors were able to be isolated in the ‘ILz:rhTRAIL/bortezomib’ group because the other tumors had almost completely regressed

To identify whether tumors would shrink with the combined treatment of ILz:rhTRAIL and bortezomib, a syngeneic mouse tumor model was generated. As the experimental time table in Fig. 3a shows, CT26 cells were subcutaneously injected into 7-week old Balb/c mice. On day 10, following the injection of CT26 cells, when average tumor volume reached around 40 mm^3^, mice were randomly grouped into four cohorts. ILz:rhTRAIL (10 μg/kg mouse) and/or bortezomib (3.8 μg/kg mouse) were administered on alternate days via tail vein, into mice belonging to the ‘ILz:rhTRAIL’, ‘bortezomib’ and ‘ILz:rhTRAIL/bortezomib’ groups. The administration interval was determined based on preliminary experiments and a previous report [40]. Tumor volume was measured every 2-days until mice were sacrificed. The average tumor volumes recorded from 10 to 18 days after injection with CT26 cells are listed in Fig. 3b. Tumors in the ‘bortezomib’ and ‘ILz:rhTRAIL/bortezomib’ groups were reduced to 50% and 30% of their respective ‘sham’ tumor volumes prior to sacrifice. The extent of tumor regression seen in vivo*,* from treatment with bortezomib only or ILz:rhTRAIL/bortezomib, was reasonable considering that in in vitro studies: around 30% of CT26 cells were killed with bortezomib and 70% of CT26 cells were killed with ILz:rhTRAIL/bortezomib. Pictures, which were taken after sacrifice, of isolated tumors from each group are shown in Fig. 3c and Additional file 4: Figure S4. In each group, seven mice were sacrificed and seven tumors were isolated. In the ‘ILz:rhTRAIL/bortezomib’ group, only five tumors were isolated, as two of the seven tumors were too small to be isolated.

### The combined treatment-induced cell death was mediated by caspase-dependent TRAIL signaling

**Fig. 4:**
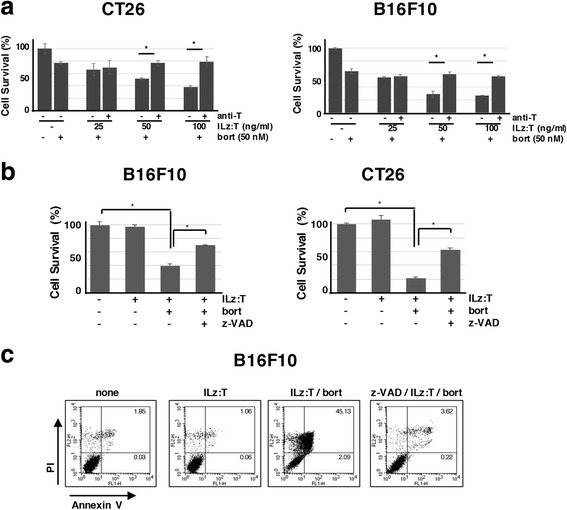
Combined treatment-induced cell death was inhibited by anti-TRAIL antibody and pan-caspase inhibitor. (**a**) After the pre-treatment of anti-TRAIL antibody (1 μg/ml) for 1 h, B16F10 and CT26 cells were treated with the indicated amount of bortezomib (50 nM) and varying amounts of ILz:rhTRAIL: ILz:T, ILz:rhTRAIL; bort, bortezomib; anti-T, anti-TRAIL antibody. Cell death was analyzed by XTT assay 24 h after treatment. (**b**) B16F10 and CT26 cells were cultured onto a 96-well plate and treated with ILz:rhTRAIL (100 ng/ml) and bortezomib (100 nM) with or without z-VAD-fmk (50 μM) pre-treatment for 1 h. After 24 h, cell death was assayed by XTT: ILz:T, ILz:rhTRAIL; bort, bortezomib; z-VAD, z-VAD-fmk. *p < 0.05 by Student’s *t*-test. (**c**) B16F10 cells were stained with propidium iodide (PI) and Annexin V using FITC Annexin V Apoptosis Detection Kit (BD Biosciences). Cells were harvested after trypsin treatment and the stained populations were analyzed by Flow cytometry (FACSCalibur™, BD Biosciences, US) using BD CellQuest™ program. The populations (%) are marked in the figures

Since cell death rates in the combined treatment were dependent upon recombinant TRAIL, we investigated whether TRAIL signaling could regulate this cell death. The combined treatment-induced cell death was found to be inhibited in CT26 and B16F10 cells when they were pre-treated with anti-TRAIL blocking antibody (Fig. 4a). The cell death rate was reduced with the addition of z-VAD-fmk, a pan-caspase inhibitor, and determined by XTT assay in CT26 and B16F10 cells (Fig. 4b). Annexin V-positive and propidium iodide (PI)-positive cells were shown to be increased with the combined treatment, but decreased with z-VAD-fmk pre-treatment, as demonstrated by flow cytometry analysis (Fig. 4c). Additionally, cell death was not inhibited by Necrostatin-1 in CT26 and B16F10 cells, although annexin V-negative and PI-positive cells were shown to increase (data not shown). The cell death seemed to be caused by necrotic cell death followed by apoptosis, because annexin V-negative and PI-positive cells, as well as annexin V-positive and PI-positive cells, were reduced by z-VAD-fmk (Fig. 4c).

**Fig. 5:**
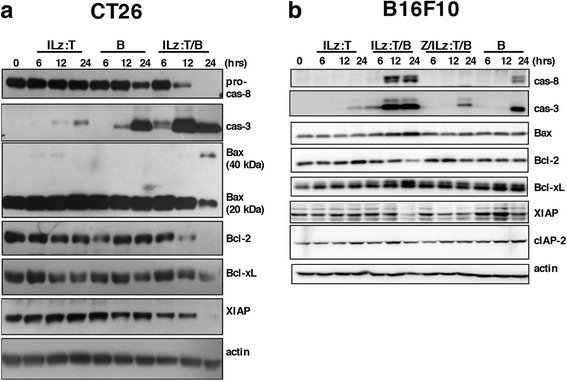
Profiling of apoptosis related proteins in CT26 and B16F10 cells after the combined treatment. ILz:rhTRAIL (100 ng/ml) and bortezomib (100 nM) were treated into CT26 (**a**) and B16F10 cells with or without pre-treatment with z-VAD-fmk: ILz:T, ILz:rhTRAIL; B, bortezomib; Z, z-VAD-fmk (**b**). After the indicated time periods, live and dead cells were harvested. Cell lysates were prepared using RIPA buffer and immunoblotting analysis was performed with appropriate antibodies, as described in ‘Materials and Methods’. Caspase-3 and -8 refer to cleaved caspases-3 and -8, respectively. Bax (40 kDa) refers to dimerized Bax. Actin was used as a loading control. *p < 0.05 by Student’s *t*-test

Since the combined treatment-induced cell death was caspase-dependent apoptosis by TRAIL, profiling of apoptosis-related proteins was performed following combined treatment in CT26 and B16F10 cells (Fig. 5). Immunoblotting analysis showed that cleaved caspase 8 and caspase 3 were detected from 12 h after the combined treatment in both cell types. Bax was noted to be increased in B16F10 cells and Bax dimer was noted to be present in CT26 cells. With the combined treatment, Bcl-2, one of the anti-apoptotic proteins which counteracts Bax, was decreased in both cell types. Another counteracting anti-apoptotic protein, Bcl-xL, was decreased in combination treated CT26 cells. Expression of XIAP (X-linked inhibitor of apoptosis protein) was down-regulated from 12 h after combination treatment in both cell types.

### Combined treatment-induced cell death was inhibited by neither ER stress inhibitors nor autophagy inhibitors

Bortezomib induces ER (endoplasmic reticulum) stress, and the proteins that induce this stress are known to increase TRAIL-sensitivity in some cells [41, 42]. Thus, ER stress related proteins were up-regulated by bortezomib treatment in CT26 and B16F10 cells, however the cell death induced by the combined treatment was not influenced by the addition of ER stress inhibitors in CT26 and B16F10 cells (Additional file 5: Figure S5). As the ER stress response is related to autophagy, LC-3 conversion was increased by the addition of bortezomib in B16F10 cells (Additional file 6: Figure S6 a). However, this expression regulation was not modulated by the combined treatment of ILz:rhTRAIL and bortezomib in the presence or absence of z-VAD-fmk. The combined treatment induced cell death was not regulated by Bafilomycin A1 (BfA1), an autophagy inhibitor, in CT26 and B16F10 cells (Additional file 6: Figure S6 b). The expression of downstream proteins in the mTOR pathway was modulated by bortezomib, but not by ILz:rhTRAIL (Additional file 6: Figure S6 c). The combined treatment induced cell death was not inhibited by the addition of mTOR inhibitors (rapamycin and torin-1) in either cell type (Additional file 6: Figure S6 d). Even though ER stress response proteins and autophagy related proteins were regulated by bortezomib in CT26 and B16F10 cells, these regulators should not be necessary to induce cell death with the combined treatment.

Since JNK has been reported to be both a positive and negative regulator of TRAIL-induced cell death, JNK activation was examined in CT26 and B16F10 cells, following the combined treatment [43–45]. Phosphorylated JNK was found to be increased in both cell types with either the combination treatment or with bortezomib alone, but not by single treatment with TRAIL. As such, SP600125 (a JNK inhibitor) was pre-treated into ILz:rhTRAIL and/or bortezomib treated cells to examine the effect of JNK on combined treatment induced cell death (Additional file 7: Figure S7). Optical densities (determined by XTT assay) in ILz:rhTRAIL and SP600125 treated CT26 and B16F10 cells were significantly reduced compared to those cells treated with ILz:rhTRAIL and bortezomib, but cell death was not observed by microscopy in ILz:rhTRAIL and SP600125 treated cells (Additional file 7: Figure S7 c). Considering the additional result that the expression and phosphorylation of JNK was not regulated by the addition of z-VAD-fmk, an inhibitor of the combined treatment-induced cell death (Additional file 7: Figure S7 a), JNK does not appear to play a regulatory role in the combined treatment-induced cell death.

## Discussion

In this report, we used three kinds of recombinant soluble TRAIL proteins to identify cell death inducing ability. Two of the recombinant TRAIL proteins were from PeproTech Korea, one made with extracellular domains of mouse TRAIL sequences (rmTRAIL), while the other was composed of human TRAIL sequences (rhTRAIL). The third, ILz:rhTRAIL, contained an extracellular domain of recombinant human TRAIL and an isoleucine zipper hexamerization motif. ILz:rhTRAIL demonstrated the strongest cell death inducing ability (Fig. 1). This was seen both in TRAIL susceptible cells, and in TRAIL resistant cells treated with the bortezomib (Fig. 2). Using these recombinant TRAIL proteins, we investigated TRAIL-induced apoptosis by single treatment with TRAIL and by the combined treatment with TRAIL and bortezomib in both TRAIL susceptible and TRAIL-resistant human and murine cells. Multiple cell types were chosen based on their susceptibility to TRAIL-induced apoptosis and their species origin. Some researchers have theorized that the TRAIL susceptibility of murine cells may be dependent upon the species origin of the TRAIL proteins. However, in this report, death in TRAIL-resistant murine cells was induced by combined treatment with ILz:rhTRAIL and bortezomib. To investigate the cell death-inducing ability of the combined treatment in transformed cells, HEK 293 and BMK cells were used. HEK293 cells are transformed human cells susceptible to TRAIL-induced apoptosis, whereas BMK cells are transformed cells resistant to TRAIL-induced apoptosis.

Although four TRAIL-resistant cells (CT26, B16F10, BMK and 4 T1) were shown to be resistant or moderately resistant to bortezomib, these cells demonstrated significant susceptibility to bortezomib when combined with recombinant TRAIL proteins. Though ILz:rhTRAIL contains human TRAIL sequences, when compared to the other recombinant TRAIL proteins used in this report, ILz:rhTRAIL showed the highest cell death inducing ability in all cell types regardless of TRAIL susceptibility and species specificity. In the combination treatment, rmTRAIL showed stronger cell death inducing ability than rhTRAIL in all three murine cell lines (CT26, B16F10 and 4 T1), but not in BMK cells. Although species specific binding of TRAIL could affect the combined treatment-induced cell death in some murine cells, the structural characteristics, rather than species specific sequences, of recombinant TRAIL must be a major determining factor in their susceptibility to TRAIL-induced cell death with the combined treatment.

The combination treatment demonstrates potential for use in in vivo tumor systems, as regression was seen in 70% of syngeneic mouse tumors when treated with equivalent amounts of ILz:rhTRAIL (10 μg/kg mouse) and bortezomib (3.8 μg/kg mouse): 100 ng/ml of ILz:rhTRAIL; 100 nM of bortezomib (Fig. 3). Because 25 nM of bortezomib and 50 ng/ml of ILz:rhTRAIL were enough to induce CT26 cell death in Fig. 2b, dose reduction of ILz:rhTRAIL and bortezomib could produce similar tumor regression effects in syngeneic tumor models. To induce specific cancer cell death efficiently, further studies concerning the the development of TRAIL responsiveness, the dose reduction of bortezomib, and the development of bortezomib substitutes for use in the combined treatment would be necessary.

The expression modulations, generally observed in TRAIL-induced cell death, were detected in the combined treatment, but not in single treatment with either TRAIL or bortezomib. Considering that cell death rates were dependent on the characteristics of recombinant TRAIL, and that the cell death observed was caspase-dependent apoptosis, the combined treatment-induced cell death could be regulated by recombinant TRAIL in the intracellular conditions provided by the proteasome inhibitor. This suggestion is supported by the result that the combined treatment-induced cell death was inhibited by pre-incubation of anti-TRAIL antibody for blocking in CT26 and B16F10 cells (Fig. 4a). Although bortezomib can regulate various intracellular signaling processes involved in cell death, these were not deemed to be the direct cause of cell death in the combined treatment. Bortezomib induced the ER stress response and modulated autophagy in cells, however, ER stress inhibition and regulation of autophagy were not shown to modulate the cell death induced by the combined treatment of ILz:rhTRAIL and bortezomib (Additional file 5: Figure S5 and Additional file 6: Figure S6).

In the experiment to investigate whether cell death signaling could be transmitted by JNK activation, cell death appeared to be regulated by SP600125 in ILz:rhTRAIL treated cells based on the comparison of optical densities by XTT assay (Additional file 7: Figure S7 b). Although the optical density of the XTT assay in ILz:rhTRAIL pre-treated with SP600125 was reduced to the optical density seen in the combined treated cells, microscopic observation demonstrated no cell death (Additional file 7: Figure S7 c). This may be due to inhibition of the reactivity of the XTT substrate by SP600125. The ER stress response and JNK activation have been reported as either positive or negative regulators of TRAIL-induced cell death in several cells; however, the combination treatment-induced cell death in B16F10 and CT26 cells was not regulated by the ER stress response, autophagy, or JNK activation [42, 45–48].

## Conclusions

In the combined treatment of recombinant TRAIL with bortezomib, the ILz:rhTRAIL containing multimerization motif showed the highest ability to induce cell death, regardless of species specificity. The cell death induced by the combined treatment was shown to be regulated by the TRAIL signaling pathway under the intracellular conditions provided by bortezomib.

## Additional files

Additional file 1: **Figure S1.** Cell death was dose-dependently increased by the addition of ILz:rhTRAIL. Cells were cultured onto 96-well plates and treated with serially increasing amount of recombinant TRAIL proteins: rmT, rmTRAIL; rhT, rhTRAIL; ILz:T, ILz:rhTRAIL. Cell survival was examined by XTT assay after 24 h. (TIFF 134 kb)

Additional file 2: **Figure S2.** Cell death-inducing abilities were analyzed in the 4 T1 cells after treatment with recombinant TRAIL proteins. Indicated amounts of recombinant TRAIL proteins were treated into 4 T1 cells and XTT assay was performed at 24 h: rmT, rmTRAIL; rhT, rhTRAIL; ILz:T, ILz:rhTRAIL. Statistical significance by two-tailed Student’s *t*-test: ***p < 0.01. (TIFF 101 kb)

Additional file 3: **Figure S3.** Cell death inducing ability of bortezomib was analyzed in TRAIL-resistant cells. Four TRAIL resistant cells (B16F10, CT26, BMK, and 4 T1) were cultured onto 96-well plates with 80% to 90% confluency and treated with increasing amounts of bortezomib, labeled as “bort.” Cell death was analyzed by XTT assay 24 h after the indicated treatment. *p < 0.05 by Student’s *t*-test, obtained after comparison with untreated control. (TIFF 103 kb)

Additional file 4: **Figure S4.** Pictures of tumors in the syngeneic mouse tumor model taken before sacrifice. Tumor volumes were measured and pictures were taken 18 days after the CT26 injection. (TIFF 2911 kb)

Additional file 5: **Figure S5.** Combined treatment-induced cell death was not inhibited by the addition of ER stress inhibitors. Cultured B16F10 (A) and CT26 cells (B) were treated with 100 nM of bortezomib for the indicated period and harvested using RIPA buffer. Immunoblotting analysis was performed using appropriate antibodies against ER stress responsive proteins: ATF6, activating transcription factor 6; CHOP, CCAAT-enhancer-binding protein homologous protein; p-PERK, phosphorylated protein kinase RNA-like Endoplasmic reticulum kinase. (C) B16F10 cells, pre-treated with various ER stress inhibitors for 3 h, were treated with ILz:rhTRAIL (100 ng/ml) and bortezomib (100 nM): ILz:T/B, combined treatment of ILz:rhTRAIL and bortezomib; 4μ8C (10 nM), IRE1 inhibitor; GSK (30 nM), PERK inhibitor (GSK2606414); sal (30 μM), selective inhibitor of eIF2α phosphorylation (salubrinal). After 36 h, live cells were stained with crystal violet. (TIFF 523 kb)

Additional file 6: **Figure S6.** Combined treatment-induced cell death was not inhibited by the addition of autophagy inhibitors. (A, B) Cultured B16F10 cells were treated with ILz:rhTRAIL (100 ng/ml) and bortezomib (100 nM) with or without z-VAD-fmk, pan-caspase inhibitor: ILz:T, ILz:rhTRAIL; B, bortezomib; Z, z-VAD-fmk. After the indicated period, cells were harvested and cell lysates were prepared for immunoblotting analysis using appropriate antibodies: LC-3, microtubule-associated protein 1A/1B-light chain 3; Atg5, autophagy protein 5; p-S6, phosphorylated ribosome protein S6; p-S6K, phosphorylated ribosomal protein S6 kinase. (C) B16F10 and CT26 cells were cultured onto a 96-well plate and treated with ILz:rhTRAIL (100 ng/ml) and/or bortezomib (100 nM) following pre-treatment with Bafilomycin A1 (BfA1) for 3 h. Cell death was analyzed by XTT assay 24 h after the ILz:rhTRAIL and/or bortezomib treatment. (D) After pre-treatment with rapamycin (100 nM) or torin-1 (1 μM) for 3 h, ILz:rhTRAIL (100 ng/ml) and bortezomib (100 nM) were treated into cells: rapa, rapamycin. Cell death was analyzed by XTT assay 24 h after the ILz:rhTRAIL and bortezomib treatment. *p < 0.05 by Student’s *t*-test. (TIFF 236 kb)

Additional file 7: **Figure S7.** Cell death was not regulated by the addition of SP600125 in ILz:rhTRAIL treated cells. (A) CT26 and B16F10 cell lysates were prepared following the ILz:rhTRAIL and/or bortezomib treatment. Immunoblotting analysis was performed using anti-JNK or anti-phosphorylated JNK antibodies: ILz:T, ILz:rhTRAIL; B, bortezomib; Z, z-VAD-fmk. (B) CT26 and B16F10 cells were cultured onto a 96 well-plate and treated with ILz:rhTRAIL and/or bortezomib with or without SP600125 pre-treatment (1μg/ml) for 3 h: T, ILz:rhTRAIL (100 ng/ml); B, bortezomib (100 nM); SP, SP600125. After 24 h of ILz:rhTRAIL and/or bortezomib treatment, XTT assay was performed. *p < 0.05 by Student’s *t*-test. (C) The morphology of ILz:rhTRAIL and/or bortezomib treated B16F10 cells was observed microscopically after 24 h: ILz:T, ILz:rhTRAIL; B, bortezomib; SP, SP600125. (TIFF 464 kb)

## Abbreviations

ATF6: Activating Transcription Factor 6
BfA1: Bafilomycin A1
CHOP: CCAAT-enhancer-binding protein Homologous Protein
ILz:rhTRAIL: isoleucine zipper hexamerization motif containing recombinant human TRAIL
JNK: c-Jun N-terminal Kinase
Nec-1: Necrostatin-1
PERK: Phosphorylated protein kinase RNA-like Endoplasmic Reticulum Kinase
TRAIL: Tumor necrosis factor Related Apoptosis Inducing Ligand
