# Supplementary figures and images for: The regulation of combined treatment-induced cell death with recombinant TRAIL and bortezomib through TRAIL signaling in TRAIL-resistant cells
Source: BMC Cancer. 2018 Apr 16;18:432. doi: 10.1186/s12885-018-4352-3 (PMC5902847; doi:10.1186/s12885-018-4352-3)

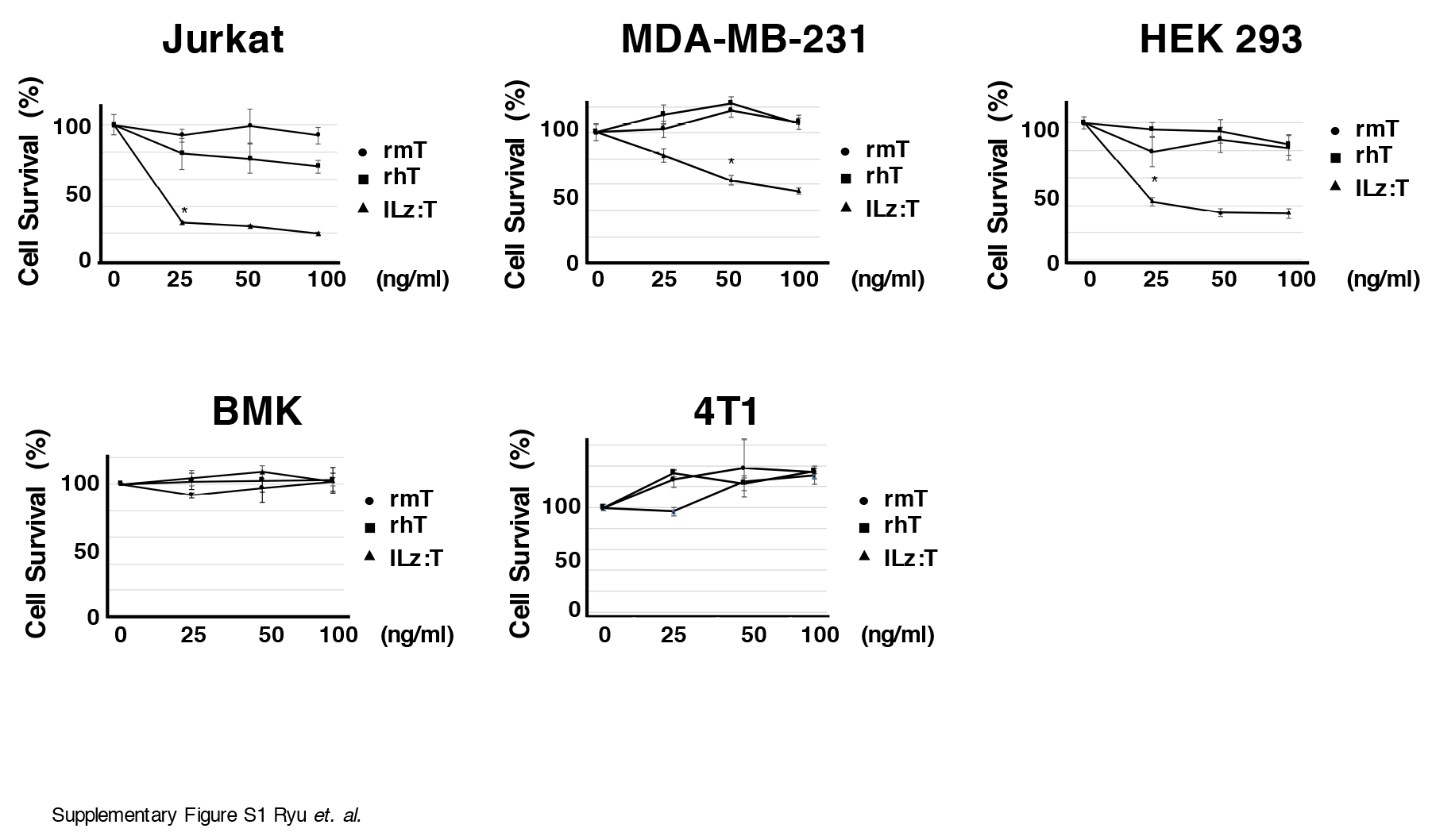

Supplement: Supplementary file 1 — Figure S1. Cell death was dose-dependently increased by the addition of ILz:rhTRAIL. Cells were cultured onto 96-well plates and treated with serially increasing amount of recombinant TRAIL proteins: rmT, rmTRAIL; rhT, rhTRAIL; ILz:T, ILz:rhTRAIL. Cell survival was examined by XTT assay after 24 h. (TIFF 134 kb) [file 12885_2018_4352_MOESM1_ESM.tif]

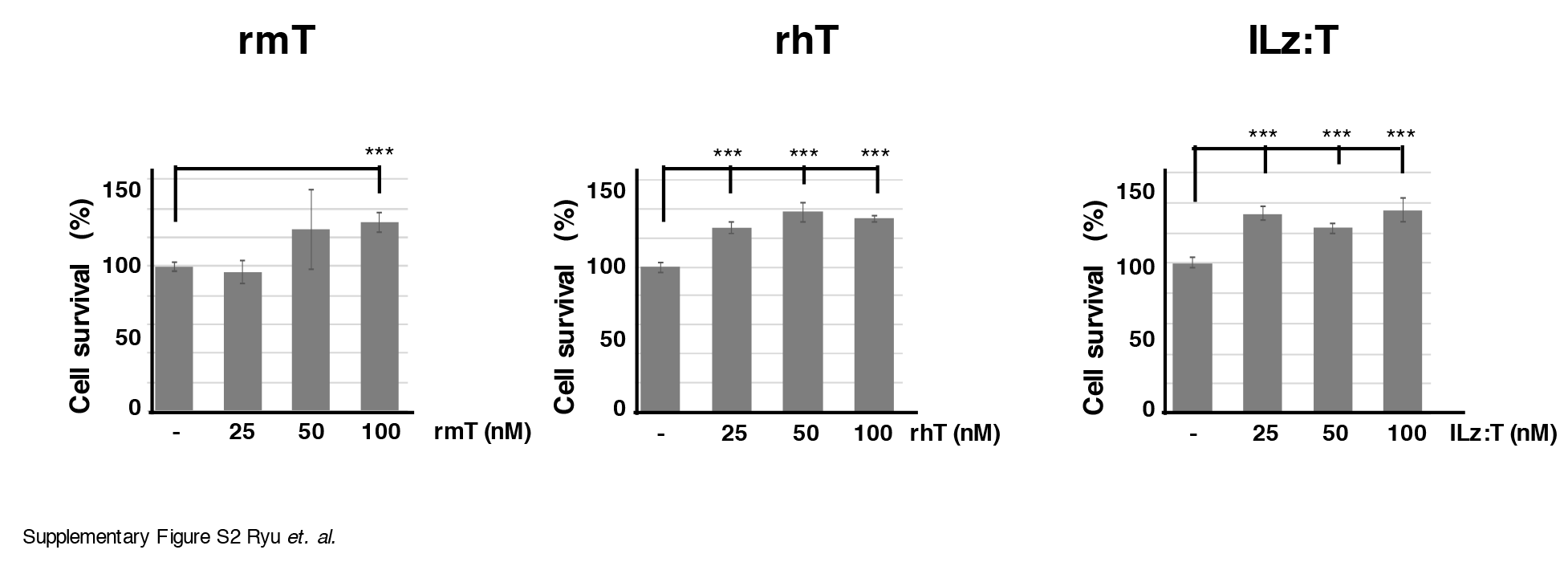

Supplement: Supplementary file 2 — Figure S2. Cell death-inducing abilities were analyzed in the 4 T1 cells after treatment with recombinant TRAIL proteins. Indicated amounts of recombinant TRAIL proteins were treated into 4 T1 cells and XTT assay was performed at 24 h: rmT, rmTRAIL; rhT, rhTRAIL; ILz:T, ILz:rhTRAIL. Statistical significance by two-tailed Student’s t-test: ***p < 0.01. (TIFF 101 kb) [file 12885_2018_4352_MOESM2_ESM.tif]

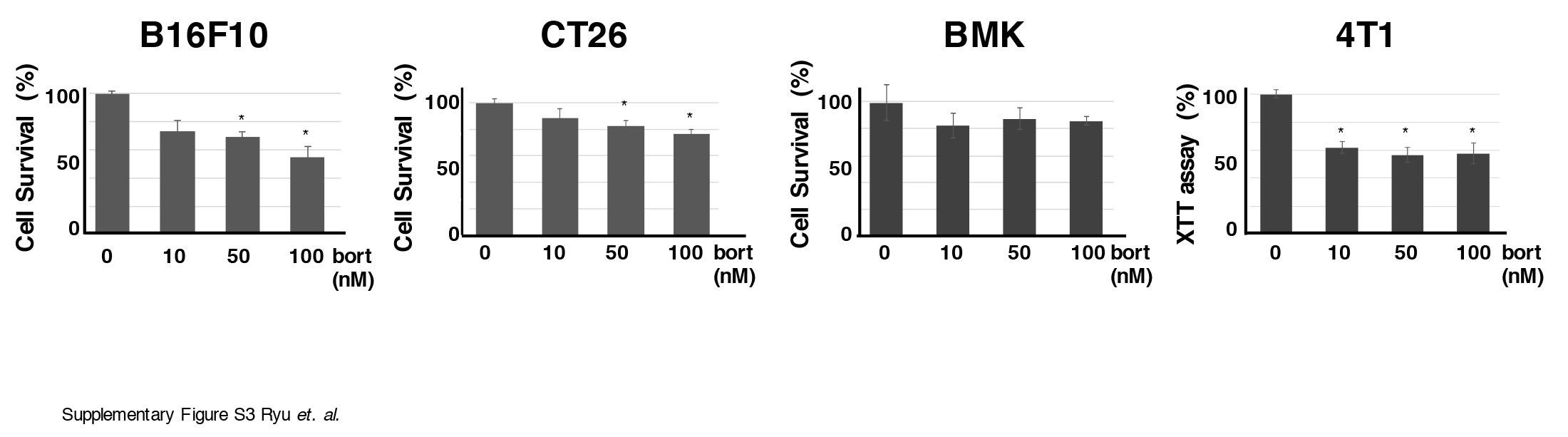

Supplement: Supplementary file 3 — Figure S3. Cell death inducing ability of bortezomib was analyzed in TRAIL-resistant cells. Four TRAIL resistant cells (B16F10, CT26, BMK, and 4 T1) were cultured onto 96-well plates with 80% to 90% confluency and treated with increasing amounts of bortezomib, labeled as “bort.” Cell death was analyzed by XTT assay 24 h after the indicated treatment. *p < 0.05 by Student’s t-test, obtained after comparison with untreated control. (TIFF 103 kb) [file 12885_2018_4352_MOESM3_ESM.tif]

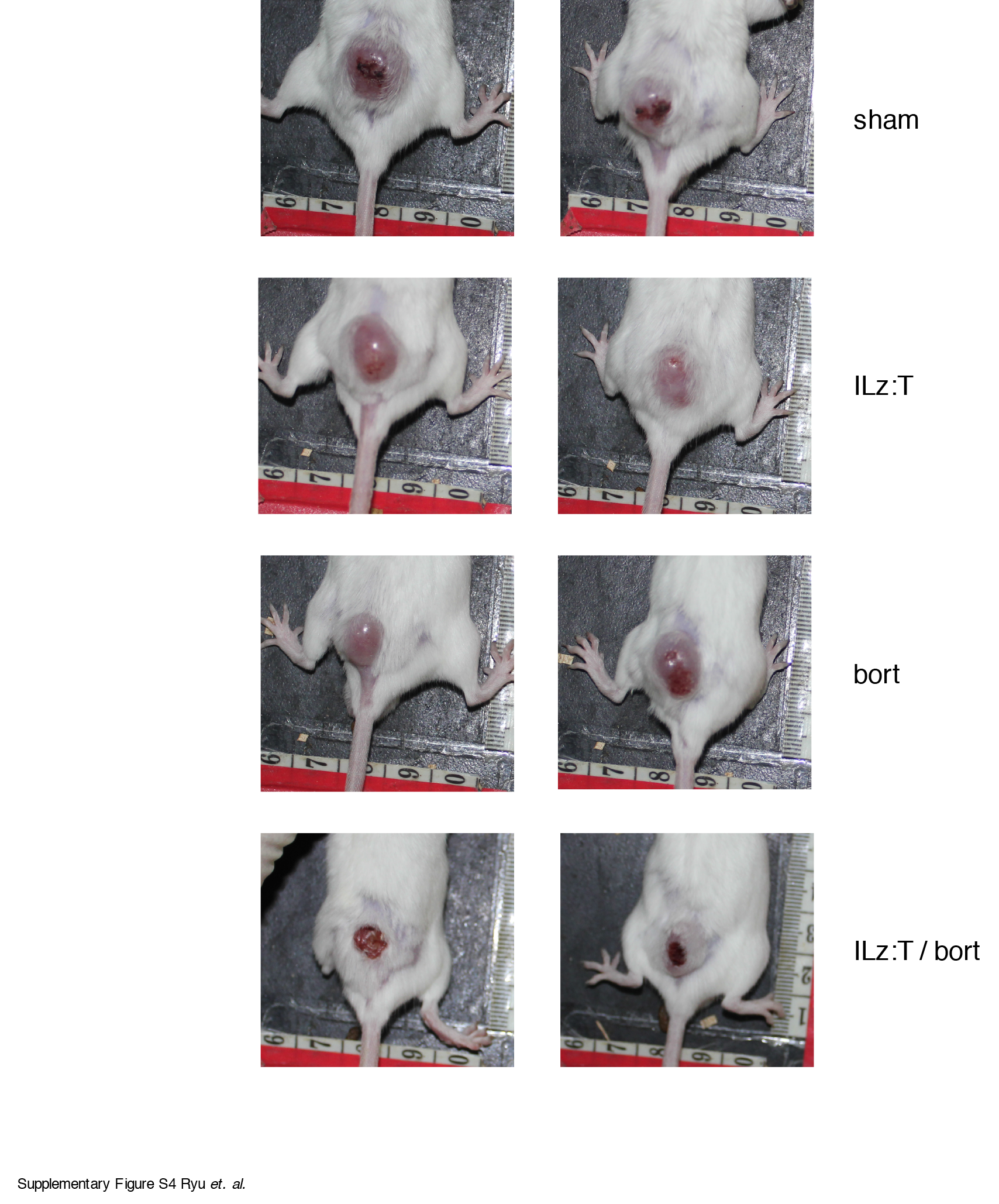

Supplement: Supplementary file 4 — Figure S4. Pictures of tumors in the syngeneic mouse tumor model taken before sacrifice. Tumor volumes were measured and pictures were taken 18 days after the CT26 injection. (TIFF 2911 kb) [file 12885_2018_4352_MOESM4_ESM.tif]

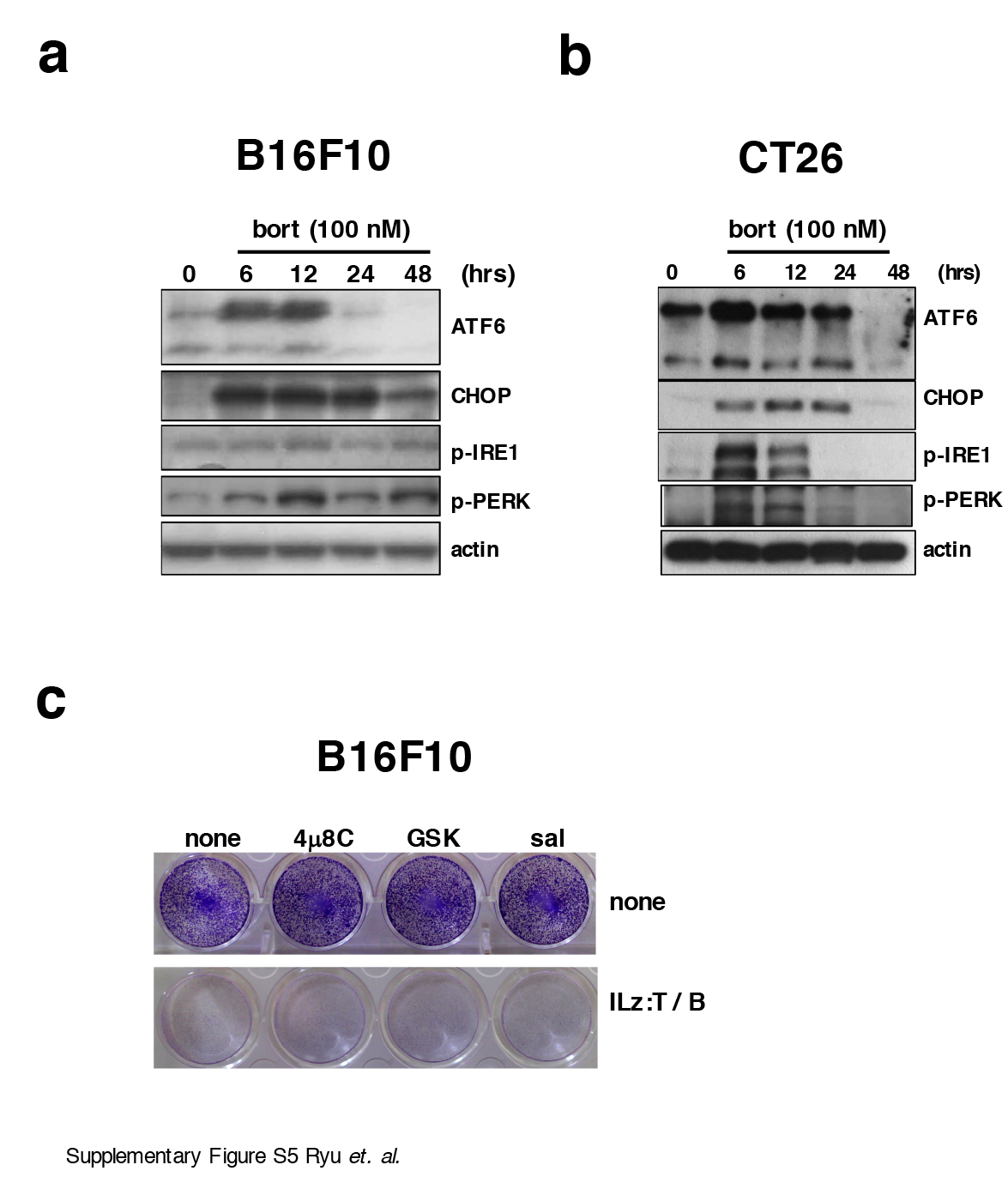

Supplement: Supplementary file 5 — Figure S5. Combined treatment-induced cell death was not inhibited by the addition of ER stress inhibitors. Cultured B16F10 (A) and CT26 cells (B) were treated with 100 nM of bortezomib for the indicated period and harvested using RIPA buffer. Immunoblotting analysis was performed using appropriate antibodies against ER stress responsive proteins: ATF6, activating transcription factor 6; CHOP, CCAAT-enhancer-binding protein homologous protein; p-PERK, phosphorylated protein kinase RNA-like Endoplasmic reticulum kinase. (C) B16F10 cells, pre-treated with various ER stress inhibitors for 3 h, were treated with ILz:rhTRAIL (100 ng/ml) and bortezomib (100 nM): ILz:T/B, combined treatment of ILz:rhTRAIL and bortezomib; 4μ8C (10 nM), IRE1 inhibitor; GSK (30 nM), PERK inhibitor (GSK2606414); sal (30 μM), selective inhibitor of eIF2α phosphorylation (salubrinal). After 36 h, live cells were stained with crystal violet. (TIFF 523 kb) [file 12885_2018_4352_MOESM5_ESM.tif]

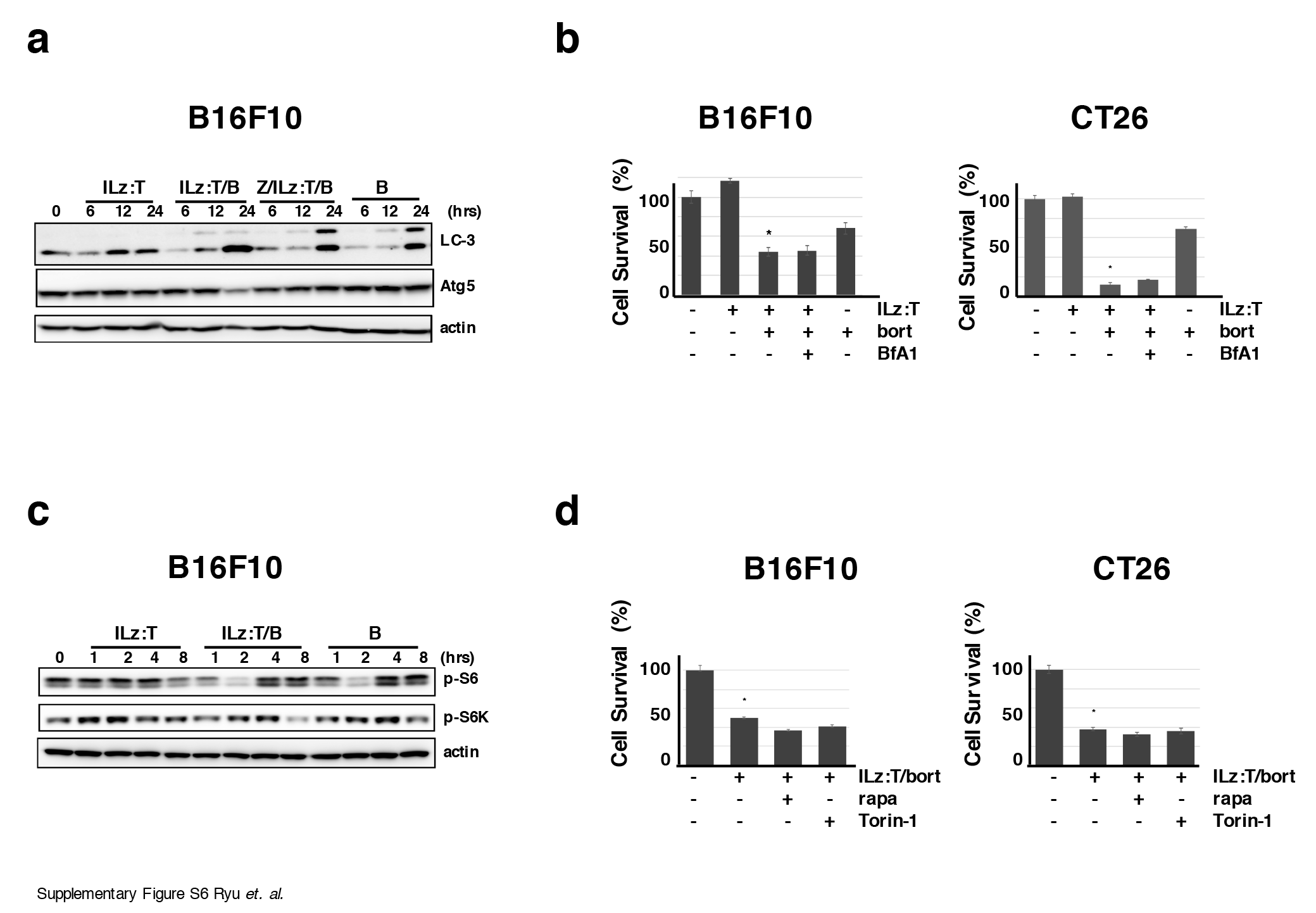

Supplement: Supplementary file 6 — Figure S6. Combined treatment-induced cell death was not inhibited by the addition of autophagy inhibitors. (A, B) Cultured B16F10 cells were treated with ILz:rhTRAIL (100 ng/ml) and bortezomib (100 nM) with or without z-VAD-fmk, pan-caspase inhibitor: ILz:T, ILz:rhTRAIL; B, bortezomib; Z, z-VAD-fmk. After the indicated period, cells were harvested and cell lysates were prepared for immunoblotting analysis using appropriate antibodies: LC-3, microtubule-associated protein 1A/1B-light chain 3; Atg5, autophagy protein 5; p-S6, phosphorylated ribosome protein S6; p-S6K, phosphorylated ribosomal protein S6 kinase. (C) B16F10 and CT26 cells were cultured onto a 96-well plate and treated with ILz:rhTRAIL (100 ng/ml) and/or bortezomib (100 nM) following pre-treatment with Bafilomycin A1 (BfA1) for 3 h. Cell death was analyzed by XTT assay 24 h after the ILz:rhTRAIL and/or bortezomib treatment. (D) After pre-treatment with rapamycin (100 nM) or torin-1 (1 μM) for 3 h, ILz:rhTRAIL (100 ng/ml) and bortezomib (100 nM) were treated into cells: rapa, rapamycin. Cell death was analyzed by XTT assay 24 h after the ILz:rhTRAIL and bortezomib treatment. *p < 0.05 by Student’s t-test. (TIFF 236 kb) [file 12885_2018_4352_MOESM6_ESM.tif]

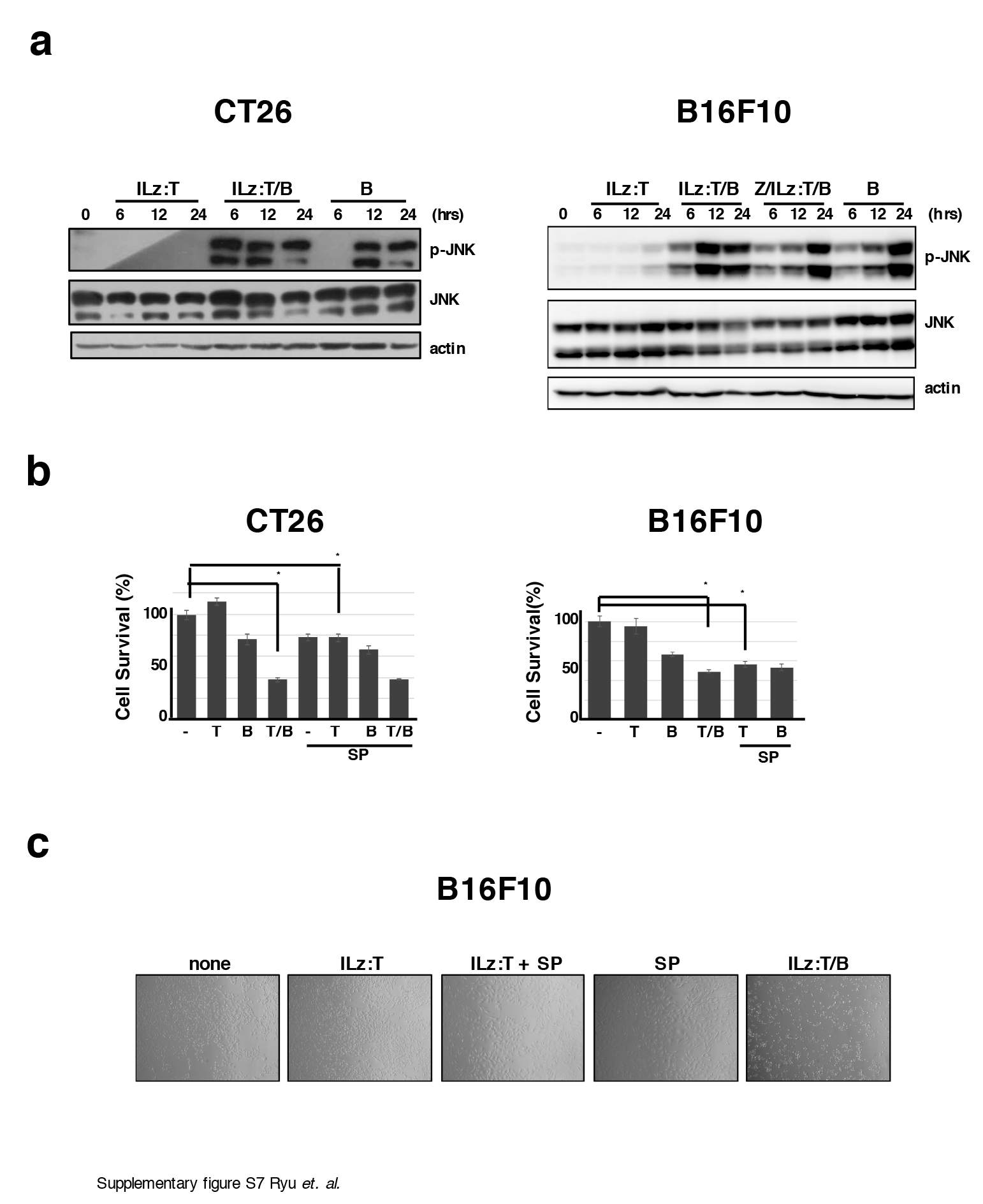

Supplement: Supplementary file 7 — Figure S7. Cell death was not regulated by the addition of SP600125 in ILz:rhTRAIL treated cells. (A) CT26 and B16F10 cell lysates were prepared following the ILz:rhTRAIL and/or bortezomib treatment. Immunoblotting analysis was performed using anti-JNK or anti-phosphorylated JNK antibodies: ILz:T, ILz:rhTRAIL; B, bortezomib; Z, z-VAD-fmk. (B) CT26 and B16F10 cells were cultured onto a 96 well-plate and treated with ILz:rhTRAIL and/or bortezomib with or without SP600125 pre-treatment (1μg/ml) for 3 h: T, ILz:rhTRAIL (100 ng/ml); B, bortezomib (100 nM); SP, SP600125. After 24 h of ILz:rhTRAIL and/or bortezomib treatment, XTT assay was performed. *p < 0.05 by Student’s t-test. (C) The morphology of ILz:rhTRAIL and/or bortezomib treated B16F10 cells was observed microscopically after 24 h: ILz:T, ILz:rhTRAIL; B, bortezomib; SP, SP600125. (TIFF 464 kb) [file 12885_2018_4352_MOESM7_ESM.tif]
